# Association between polyunsaturated fatty acids and depression in women with infertility: a cross-sectional study based on the National Health and Nutrition Examination Survey

**DOI:** 10.3389/fpsyt.2024.1345815

**Published:** 2024-07-02

**Authors:** Yan Hong, Xia Jin, Lihong Shi

**Affiliations:** ^1^ Department of Traditional Chinese Medicine, Tongji Hospital, School of Medicine, Tongji University, Shanghai, China; ^2^ Department of Gynaecology and Obstetrics, Tongji Hospital, School of Medicine, Tongji University, Shanghai, China; ^3^ Department of Reproductive Medicine, Tongji Hospital, School of Medicine, Tongji University, Shanghai, China

**Keywords:** polyunsaturated fatty acids, omega-3 PUFA, omega-6 PUFA, depression, infertility

## Abstract

**Background:**

Identifying possible influencing factors is crucial for the depression symptoms of women experiencing infertility. This study aims to explore the association between polyunsaturated fatty acids (PUFAs) and the odds of depression symptoms in women experiencing infertility.

**Methods:**

This is a cross-sectional study based on the National Health and Nutrition Examination Survey (NHANES). PUFA intake was obtained through a 24-h dietary recall interview. Depression symptoms were defined using the Patient Health Questionnaire-9 (PHQ-9) with a score of ≥10 points or as taking antidepressants. The association between PUFA and depression was assessed using a logistic regression model by calculating the odds ratio (OR) with 95% confidence interval (CI). Subgroup analysis was carried out based on menopausal status and female hormone use.

**Results:**

There were 725 participants included for analysis. After adjusting the covariables, lower odds of depression symptoms were found in patients with the intake of omega-3 PUFA (OR = 0.48, 95% CI: 0.24–0.96) and omega-6 PUFA (OR = 0.24, 95% CI: 0.14–0.42) in the second tertile (T2) in comparison to the first tertile (T1). The intake of α-linolenic (ALA) (OR = 0.48, 95% CI: 0.23–0.97) and linoleic acid (OR = 0.24, 95% CI: 0.14–0.41) in T2 was also found to be related to the reduced odds of depression symptoms in comparison to T1.

**Conclusions:**

Our findings suggest a potential association between moderate omega-3 and omega-6 PUFA intake and a reduced risk of depression symptoms in women experiencing infertility. This implies that clinicians might find it useful to consider dietary advice that includes PUFA-rich foods as part of a broader strategy to address mental health in this patient group. However, further research is needed to confirm these preliminary findings and to establish the optimal levels of PUFA intake for mental health benefits.

## Introduction

Infertility is commonly defined as failure to conceive after attempting to conceive for more than 12 months (including women who subsequently become pregnant) (1) and affects an estimated 15% of couples globally (2). Women with infertility may suffer from great psychosocial stress, and 31%–58% of them are experiencing depression (3, 4). The risk of depression increases with the increase in the duration of infertility, which affects the physical and mental health and quality of life in women with infertility (5). Therefore, it is crucial to pay attention to the depression situation of infertile women and identify possible influencing factors.

Increasing evidence shows the significance of nutrition in female fertility, and a key mechanism by which nutrition may affect reproductive function is the regulatory effect of many nutrients and non-nutrient food components on inflammatory processes (6). Polyunsaturated fatty acids (PUFAs), characterized by their multiple double bonds, predominantly consist of omega-3 and omega-6 fatty acids (7). Generally, omega-3 fatty acids are thought to play an anti-inflammatory role, and omega-6 fatty acids exert a pro-inflammatory effect (7); therefore, the ratio of omega-3 to omega-6 (omega-3/omega-6) is also important for inflammation balance. Some studies have shown that the occurrence and development of infertility and depression are both related to pro-inflammatory cytokines (6, 8). Wang et al. observed that an increased consumption of docosahexaenoic acid (DHA) within omega-3 fatty acids correlated with a reduced risk of infertility; conversely, a greater ratio of omega-6 to omega-3 fatty acids was linked to an elevated risk of infertility (7). In a systematic review and meta-analysis, for perinatal women with depression, there were significantly lower levels of total omega-3 PUFAs and increased omega-6 to omega-3 ratio (9). The NuPED study showed that higher omega-6 to omega-3 PUFA ratios early in pregnancy were associated with higher odds for depression at 12 months postpartum (10). Whether there is a different relationship between omega-3/omega-6 fatty acid consumption and depression in women experiencing infertility needs to be considered. In addition, several studies have indicated the different impacts of different types of PUFAs on infertility or depression. A previous study suggested that omega-3 fatty acids are effective in alleviating depression symptoms, and supplementing two main types of omega-3 fatty acids, eicosapentaenoic acid (EPA) and DHA, has also been observed to be effective in improving depression (11, 12). In a study conducted among Japanese women, there was no significant association between EPA, DHA, and n−3 PUFA intake during the third trimester and postpartum depression (1 and 6 months after delivery) (13). In another study, DHA was slightly related to the risk of infertility; however, women with α-linolenic (ALA) intake presented with a relatively higher risk of primary infertility (7). However, the association of various types of PUFAs with the risk of depression in infertile women has not been reported.

In this study, we aimed to explore the association between omega-3, omega-6, and omega-3/omega-6 with the risk of depression symptoms in women experiencing infertility. We also investigated the specific associations between various subtypes of omega-3 and omega-6 PUFAs and the risk of depression symptoms.

## Methods

### Study design and data source

This is a cross-sectional study, and data were extracted from the 2013–2020 National Health and Nutrition Examination Survey (14), which has a complex, multistage, stratified design. NHANES combined interviews and physical examinations, with interviews including questions related to demographics, socioeconomics, diet, and health and examinations including medical, dental, and physiological measurements and laboratory tests. The protocol of NHANES has been approved by the National Center for Health Statistics (NCHS) Research Ethics Review Board, and each participant has provided informed consent during the survey. Therefore, this study did not require an ethical review from Tongji Hospital, School of Medicine, Tongji University.

### Study population

Participants meeting the following criteria were included: 1) diagnosed as infertile and 2) age ≥18 years old. Participants meeting one of the following criteria were excluded: 1) missing complete information of PUFA intake; 2) missing information for depression assessment; 3) unusually low or high total energy intake (<500 kcal/day or >5,000 kcal/day); and (4) missing information of key covariables of PUFA intake, depression assessment, marital status, age at menarche, sleep duration, and body mass index (BMI).

Information on infertility was analyzed using data from the NHANES spanning from 2013 to 2020. Reproductive health-related information was extracted from the Reproductive Health Questionnaire (RHQ). Infertility was defined based on the responses to RHQ074—”Tried for a year to become pregnant?” with an answer of “yes” indicating infertility, or by a “yes” to either RHQ074—”Tried for a year to become pregnant?” or RHQ076—”Seen a doctor because unable to become pregnant?”. The distinction between primary and secondary infertility was made based on whether the individual had ever been pregnant.

PUFA intake was obtained through a 24-h dietary recall interview. NHANES requested participants to perform two 24-h dietary recalls, with the first one performed by in-person interview and the second one performed by telephone. Omega-3 PUFAs included ALA, stearidonic acid, EPA, docosapentaenoic acid (DPA), and DHA. Omega-6 PUFAs in this study included linoleic acid and arachidonic acid (AA). This study used PUFA intake data on the first day, and the data were classified according to the tertiles.

Depression symptoms were defined using the Patient Health Questionnaire-9 (PHQ-9) with a score of ≥10 points or as taking antidepressants. The PHQ-9 included the following items: 1) anhedonia, 2) depressed mood, 3) sleep disturbance, 4) fatigue, 5) appetite changes, 6) low self-esteem, 7) concentration problems, 8) psychomotor disturbances, and 9) suicidal ideation. The total score ranged from 0 to 27, with a total score of ≥10 points defining depression (15).

### Data extraction

The data were extracted, including demographic characteristics [age, race, educational level, poverty-to-income ratio (PIR), marital status, smoking, alcohol drinking, physical activity, age at menarche, menopausal status, ever pregnant, ovariectomy, hysterectomy, sleep duration], comorbidities [hypertension, diabetes, dyslipidemia, cardiovascular disease (CVD), pelvic infection], medicine use (female hormone use), physical examination (BMI), and dietary intake (total energy, total PUFA, omega-3 PUFA, ALA, stearidonic acid, EPA, DPA, DHA, omega-6 PUFA, linoleic acid, AA).

Hypertension was defined as individuals with systolic blood pressure ≥140 mmHg or diastolic blood pressure ≥90 mmHg or self-reported hypertension or taking antihypertensive drugs (16). Diabetes was defined as fasting blood glucose ≥7.0 mmol/L or glycohemoglobin A1c (HbA1c) ≥6.5% or self-reported diabetes or receiving hypoglycemic treatment (17).

### Statistical analysis

The appropriate sample weights provided by NHANES were used to weigh the data in this study considering the complex sampling design. The masked variance unit pseudo-primary sampling unit was sdmvpsu, and the masked variance unit pseudo-stratum was sdmvstra. Confidence interval (CI) was applied to evaluate the reliability of an estimate. A set of weights WTDRD1 was the 2-year sample weight for dietary day 1, and WTDRD1PP was the dietary day 1 sample weight. The final weighting was calculated as follows: for the years 2013–2014 and 2015–2016, the weighting used was 2/7.2 * WTDRD1; for the years 2017–2020, the weighting was 3.2/7.2 * WTDRD1PP. Mean (standard error, SE) was calculated for continuous data, and the *t*-test was used to compare the differences. Number and percentage [*n* (%)] were used for the categorical data, and the chi-squared test was used to compare the differences.

The weighted univariate and multivariable logistic regression models were used to calculate the odds ratio (OR) with 95% CI to assess the association between PUFA (omega-3 PUFA, ALA, stearidonic acid, EPA, DPA, DHA, omega-6 PUFA, linoleic acid, AA) and depression symptoms. Based on the multivariable logistic regression models, two models were constructed: model 1 (unadjusted model) and model 2 (adjusted for covariates). The covariates were selected using the logistic regression model. Variates with statistical significance (*P* < 0.05) in the univariate model were included in the multivariable model. To avoid overfitting, a backward stepwise regression analysis was employed to select which of the variables should be included in a regression model. Additionally, the Akaike information criterion (AIC) was utilized to evaluate the goodness of fit of the models. A lower AIC value indicates a better-fitting model. The importance of variables (ALA, stearidonic acid, EPA, DPA, DHA, linoleic acid, and AA) was assessed using Gini importance, which was computed from the random forest model. Subgroup analysis was conducted according to menopausal status and female hormone use. The statistical analyses were conducted using SAS 9.4 (SAS Institute Inc., Cary, NC, USA) and R version 4.2.3 (R Foundation for Statistical Computing, Vienna, Austria). *P <*0.05 was considered as statistically significant.

## Results

### Selection and characteristics of the study population

**Figure 1:**
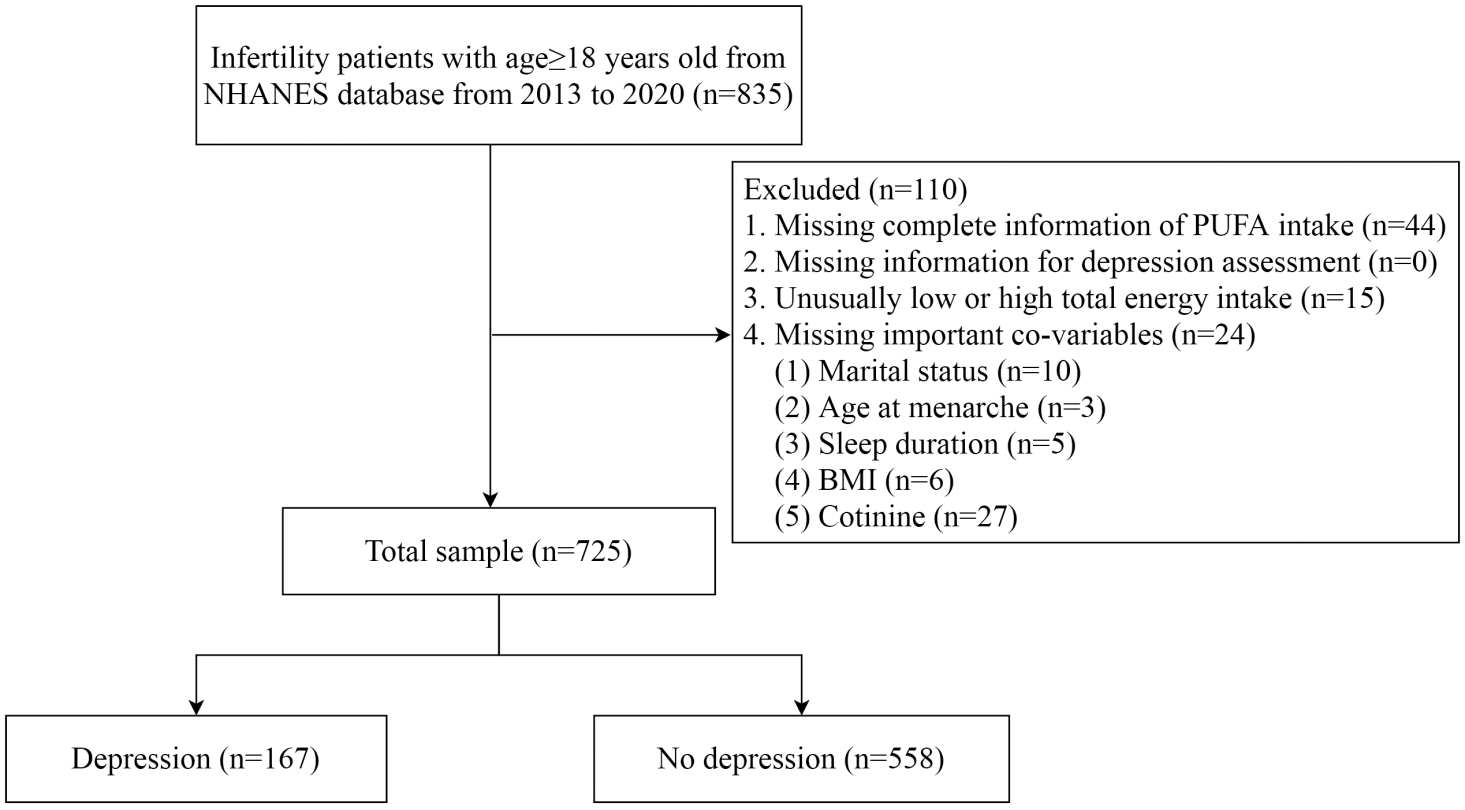
The flowchart of selecting participants.

A total of 835 women aged ≥18 years old experienced infertility based on NHANES 2013–2020. Based on the inclusion and exclusion criteria, 725 patients were included for analysis, with 167 patients in the depression group and 558 patients in the non-depression group (**Figure 1**).

Their mean age was 41.82 ± 0.61 years, and 71.09% obtained an education level of above high school. There was significance in age, PIR, marital status, smoking, alcohol drinking, physical activity, ovariectomy, hysterectomy, hypertension, diabetes, dyslipidemia, CVD, pelvic infection, female hormone use, sleep duration, and BMI between the non-depression group and the depression group. The characteristics of the included patients are shown in **Table 1**.

**Table 1:** The characteristics of the included participants.

Variables	Total (*n* = 725)	Non-depression (*n* = 558)	Depression (*n* = 167)	Statistics	*P*
Age, years, mean ± SE	41.82 ± 0.61	40.78 ± 0.74	44.68 ± 1.09	*t* = 2.818	0.007
Race, *n* (%)				χ² = 2.104	0.114
Non-Hispanic White	291 (62.05)	205 (59.19)	86 (69.86)		
Non-Hispanic Black	159 (12.90)	124 (13.88)	35 (10.23)		
Mexican American	91 (9.02)	76 (10.28)	15 (5.58)		
Others	184 (16.03)	153 (16.65)	31 (14.33)		
Educational level, *n* (%)				χ² = 1.482	0.233
Less than high school	91 (9.06)	6 5(7.70)	26 (12.76)		
High school	139 (19.85)	109 (19.15)	30 (21.79)		
Above high school	495 (71.09)	384 (73.15)	111 (65.45)		
PIR, *n* (%)				χ² = 3.590	0.034
<1	120 (11.07)	80 (9.42)	40 (15.56)		
≥1	548 (82.68)	430 (83.13)	118 (81.44)		
Unknown	57 (6.25)	48 (7.45)	9 (3.00)		
Marital status, *n* (%)				χ² = 23.174	<0.001
Married and living with a partner	520 (74.55)	427 (81.35)	93 (55.91)		
Never married, divorced, separated, and widowed	205 (25.45)	131 (18.65)	74 (44.09)		
Smoking, *n* (%)				χ² = 7.165	0.010
No	468 (62.82)	385 (67.22)	83 (50.78)		
Yes	257 (37.18)	173(32.78)	84 (49.22)		
Alcohol drinking, *n* (%)				χ² = 3.252	0.029
Never	162 (17.15)	139 (19.47)	23 (10.79)		
Former	153 (20.80)	115 (20.31)	38 (22.14)		
Continuous	346 (54.48)	263 (54.87)	83 (53.41)		
Unknown	64 (7.57)	41 (5.35)	23 (13.66)		
Physical activity, *n* (%)				χ² = 6.045	0.017
Low level	512 (65.60)	386 (62.27)	126 (74.72)		
High level	213 (34.40)	172 (37.73)	41 (25.28)		
Age at menarche, years, mean ± SE	12.62 ± 0.10	12.59 ± 0.11	12.70 ± 0.16	*t* = 0.604	0.549
Menopausal status, *n* (%)				χ² = 0.000	0.982
No	622 (83.65)	483 (83.62)	139 (83.73)		
Yes	103 (16.35)	75 (16.38)	28 (16.27)		
Ever pregnant, *n* (%)				χ² = 3.485	0.067
No	116 (14.30)	96 (16.13)	20 (9.28)		
Yes	609 (85.70)	462 (83.87)	147 (90.72)		
Ovariectomy, *n* (%)				χ² = 20.070	< 0.001
No	679 (92.22)	536 (96.53)	143 (80.40)		
Yes	46 (7.78)	22 (3.47)	24 (19.60)		
Hysterectomy, *n* (%)				χ² = 21.331	<0.001
No	631 (87.01)	502 (92.33)	129 (72.41)		
Yes	94 (12.99)	56 (7.67)	38 (27.59)		
Hypertension, *n* (%)				χ² = 14.543	<0.001
No	461 (66.41)	376 (71.94)	85 (51.27)		
Yes	264 (33.59)	182 (28.06)	82 (48.73)		
Diabetes, *n* (%)				χ² = 8.432	0.005
No	611 (85.97)	481 (89.22)	130 (77.07)		
Yes	114 (14.03)	77 (10.78)	37 (22.93)		
Dyslipidemia, *n* (%)				χ² = 7.031	0.010
No	291 (36.93)	241 (40.76)	50 (26.45)		
Yes	434 (63.07)	317 (59.24)	117 (73.55)		
CVD, *n* (%)				χ² = 15.310	<0.001
No	695 (95.53)	542 (97.53)	153 (90.03)		
Yes	30 (4.47)	16 (2.47)	14 (9.97)		
Pelvic infection, *n* (%)				χ² = 13.293	0.001
No	655 (90.45)	514 (94.00)	141 (80.72)		
Yes	70 (9.55)	44 (6.00)	26 (19.28)		
Female hormone use, *n* (%)				χ² = 16.619	<0.001
No	599 (81.91)	476 (87.15)	123 (67.55)		
Yes	126 (18.09)	82 (12.85)	44 (32.45)		
Sleep duration, hours, *n* (%)				χ² = 3.189	0.047
<7	214 (27.18)	160 (26.94)	54 (27.87)		
7–9	457 (66.65)	363 (68.53)	94 (61.49)		
>9	54 (6.17)	35 (4.53)	19 (10.64)		
BMI, kg/m^2^, *n* (%)				χ² = 5.555	0.005
<25	191 (27.58)	163 (31.92)	28 (15.73)		
25–30	154 (22.20)	124 (22.39)	30 (21.65)		
≥30	380 (50.22)	271(45.69)	109 (62.62)		
Total energy, kcal, mean ± SE	1,903.69 ± 36.00	1,934.15 ± 37.84	1,820.25 ± 75.09	t = −1.418	0.162
Total PUFA, g, mean ± SE	18.11 ± 0.62	18.41 ± 0.72	17.30 ± 1.23	t = −0.784	0.436
Omega-3 PUFA, g, mean ± SE	1.84 ± 0.08	1.90 ± 0.09	1.68 ± 0.13	t = −1.481	0.145
ALA, g, mean ± SE	1.72 ± 0.07	1.78 ± 0.09	1.56 ± 0.13	t = −1.514	0.136
Stearidonic acid, g, mean ± SE	0.01 ± 0.00	0.01 ± 0.00	0.01 ± 0.00	t = 1.423	0.161
EPA, g, mean ± SE	0.03 ± 0.00	0.03 ± 0.00	0.02 ± 0.01	t = −0.597	0.553
DPA, g, mean ± SE	0.02 ± 0.00	0.02 ± 0.00	0.02 ± 0.00	t = −0.746	0.459
DHA, g, mean ± SE	0.06 ± 0.01	0.06 ± 0.01	0.05 ± 0.02	t = −0.045	0.964
Omega-6 PUFA, g, mean ± SE	16.13 ± 0.57	16.36 ± 0.66	15.48 ± 1.11	t = −0.686	0.495
Linoleic acid, g, mean ± SE	16.00 ± 0.56	16.23 ± 0.66	15.35 ± 1.10	t = −0.692	0.492
AA, g, mean ± SE	0.13 ± 0.01	0.13 ± 0.01	0.14 ± 0.01	t = 0.220	0.826
Omega-3/omega-6, score, mean ± SE	0.13 ± 0.01	0.12 ± 0.01	0.13 ± 0.02	t = 0.409	0.684

PIR, poverty-to-income ratio; CVD, cardiovascular disease; BMI, body mass index; PUFA, polyunsaturated fatty acid; ALA, α-linolenic; EPA, eicosapentaenoic acid; DPA, docosapentaenoic acid; DHA, docosahexaenoic acid; AA, arachidonic acid.

### Association between PUFA and depression in women experiencing infertility

**Supplementary Table S1** shows that race, marital status, alcohol drinking, physical activity, ovariectomy, diabetes, pelvic infection, and female hormone use were identified as covariables. At the same time, considering that age, energy, and total PUFA may have a great impact on the results, they are also included as covariables. After adjusting these covariates, we found that the intake of omega-3 PUFA in the second tertile (T2) was associated with lower odds of depression (OR = 0.48, 95% CI: 0.24–0.96) in comparison to the first tertile (T1). A similar result was found in omega-6 PUFA: there was a significant association between omega-6 PUFA intake in T2 and decreased risk of depression (OR = 0.24, 95% CI: 0.14–0.42) (**Table 2**).

**Table 2:** Association between PUFA and depression in infertility women.

Variables	Sample size	Model 1		Model 2	
		OR (95% CI)	*P*	OR (95% CI)	*P*
Omega-3 PUFA					
T1 (<1.150)	66/236	Ref		Ref	
T2 (1.150–1.872)	45/226	0.57 (0.30–1.07)	0.081	0.48 (0.24–0.96)	0.038
T3 (≥1.872)	56/263	0.49 (0.29–0.83)	0.009	0.35 (0.12–1.04)	0.057
Omega-6 PUFA					
T1 (<10.346)	64/219	Ref		Ref	
T2 (10.346–17.908)	40/241	0.25 (0.13–0.46)	<0.001	0.24 (0.14–0.42)	< 0.001
T3 (≥17.908)	63/265	0.55 (0.33–0.91)	0.022	0.52 (0.21–1.31)	0.159
Omega-3/omega-6					
T1 (<0.097)	60/235	Ref		Ref	
T2 (0.097–0.125)	57/249	0.57 (0.32–0.99)	0.048	0.60 (0.35–1.03)	0.063
T3 (≥0.125)	50/241	0.68 (0.36–1.27)	0.220	0.60 (0.31–1.18)	0.134

Model 1: unadjusted model.

Model 2: adjusted for age, race, marital status, alcohol drinking, physical activity, ovariectomy, diabetes, pelvic infection, female hormone use, total energy, and total PUFA.

Ref, reference; OR, odds ratio; CI, confidence interval; PUFA, polyunsaturated fatty acids.

### Association between different components of PUFA and depression in women experiencing infertility

**Figure 2:**
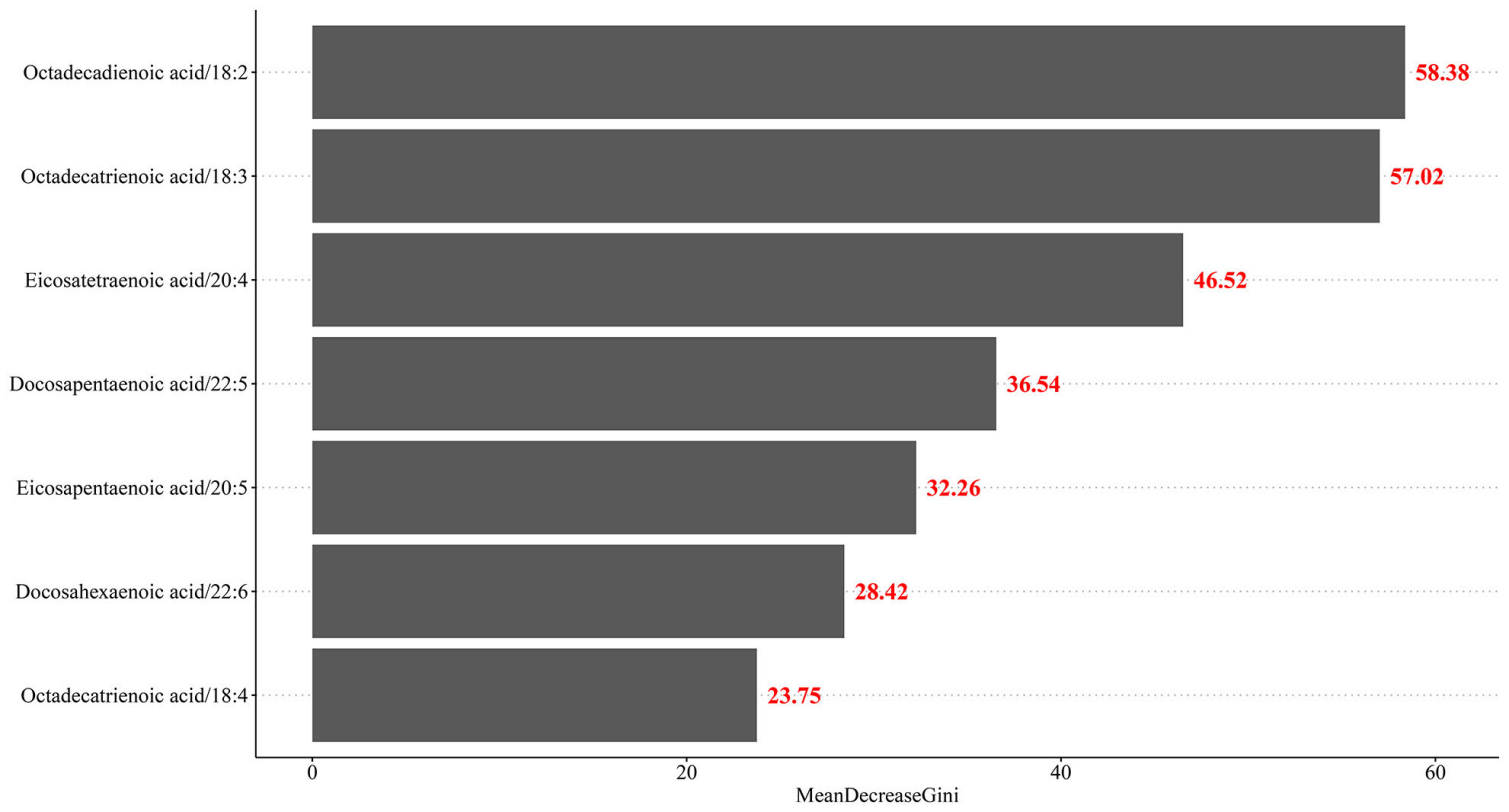
The importance of different components of PUFA.

After adjusting for age, race, marital status, alcohol drinking, physical activity, ovariectomy, diabetes, pelvic infection, female hormone use, total energy, and total PUFA, the intake of ALA in T2 and T3 was found to be related to the reduced odds of depression (T2: OR = 0.48, 95% CI: 0.23–0.97; T3: OR = 0.20, 95% CI: 0.08–0.49) in comparison to T1. The intake of linoleic acid in T2 had a relationship with the odds of depression (OR = 0.24, 95% CI: 0.14–0.41) in comparison to T1 (**Table 3**). **Figure 2** indicates that moderate intake of PUFAs, particularly linoleic acid and ALA, was associated with a lower likelihood of depression.

**Table 3:** Association between different components of PUFA and depression.

Variables	Sample size	Model 1		Model 2	
		OR (95% CI)	*P*	OR (95% CI)	*P*
ALA					
T1 (<1.042)	68/233	Ref		Ref	
T2 (1.042–1.742)	45/227	0.58 (0.30–1.11)	0.098	0.48 (0.23–0.97)	0.043
T3 (≥1.742)	54/265	0.43 (0.25–0.73)	0.003	0.20 (0.08–0.49)	0.001
Stearidonic acid					
0	58/292	Ref		Ref	
<0.003	47/183	1.54 (0.74–3.21)	0.244	1.14 (0.57–2.29)	0.700
≥0.003	62/250	1.11 (0.59–2.08)	0.748	1.07 (0.52–2.24)	0.845
EPA					
T1 (<0.004)	48/180	Ref		Ref	
T2 (0.004–0.012)	66/289	0.69 (0.33–1.43)	0.308	0.90 (0.43–1.89)	0.782
T3 (≥0.012)	53/256	0.52 (0.29–0.95)	0.034	0.63 (0.31–1.28)	0.197
DPA					
T1 (<0.009)	51/195	Ref		Ref	
T2 (0.009–0.022)	60/267	0.58 (0.30–1.12)	0.104	0.64 (0.33–1.23)	0.173
T3 (≥0.022)	56/263	0.48 (0.28–0.83)	0.010	0.57 (0.28–1.17)	0.122
DHA					
T1 (<0.004)	59/222	Ref		Ref	
T2 (0.004–0.022)	48/229	0.71 (0.36–1.42)	0.328	1.01 (0.48–2.10)	0.988
T3 (≥0.022)	60/274	0.79 (0.43–1.46)	0.452	1.13 (0.57–2.24)	0.720
Linoleic acid					
T1 (<10.263)	64/219	Ref		Ref	
T2 (10.263–17.621)	39/240	0.24 (0.13–0.44)	<0.001	0.24 (0.14–0.41)	<0.001
T3 (≥17.621)	64/266	0.56 (0.33–0.93)	0.027	0.57 (0.22–1.46)	0.232
AA					
T1 (<0.067)	53/221	Ref		Ref	
T2 (0.067–0.142)	48/231	0.70 (0.39–1.27)	0.232	0.79 (0.40–1.58)	0.501
T3 (≥0.142)	66/273	0.88 (0.50–1.56)	0.654	1.33 (0.72–2.47)	0.354

Ref, reference; OR, odds ratio; CI, confidence interval; PUFA, polyunsaturated fatty acid; ALA, α-linolenic; EPA, eicosapentaenoic acid; DPA, docosapentaenoic acid; DHA, docosahexaenoic acid; AA, arachidonic acid.

Model 1: unadjusted model.

Model 2: adjusted for age, race, marital status, alcohol drinking, physical activity, ovariectomy, diabetes, pelvic infection, female hormone use, total energy, and total PUFA.

### Association between PUFA and depression in women experiencing infertility at menopausal status or with female hormone use

**Table 4** shows that the intake of omega-3 PUFA in T2 was associated with decreased odds of depression in women experiencing infertility without female hormone use (OR = 0.34, 95% CI: 0.15–0.81). The intake of ALA in T3 had a relationship with the lower odds of depression in women experiencing infertility at menopausal status (OR = 0.01, 95% CI: 0.00–0.85), not at menopausal status (OR = 0.28, 95% CI: 0.11–0.68) and without female hormone use (OR = 0.15, 95% CI: 0.06–0.42). The T2 intake of omega-6 PUFA and linoleic acid was related to the lower odds of depression in women with infertility who are experiencing menopause and those who are not or those who are without female hormone use (all *P* < 0.05).

**Table 4:** Subgroup analysis based on menopausal status and female hormone use.

Subgroups	Menopausal status				Female hormone use			
	No		Yes		No		Yes	
	OR (95% CI)	*P*	OR (95% CI)	*P*	OR (95% CI)	*P*	OR (95% CI)	*P*
Omega-3 PUFA								
T1 (<1.150)	Ref		Ref		Ref		Ref	
T2 (1.150–1.872)	0.54 (0.28–1.04)	0.063	0.33 (0.02–5.34)	0.331	0.34 (0.15–0.81)	0.015	1.40 (0.37–5.31)	0.580
T3 (≥1.872)	0.53 (0.19–1.52)	0.229	0.01 (0.00–0.64)	0.038	0.28 (0.08–0.97)	0.045	0.32 (0.02–4.87)	0.366
ALA								
T1 (<1.042)	Ref		Ref		Ref		Ref	
T2 (1.042–1.742)	0.54 (0.28–1.06)	0.071	0.33 (0.01–8.41)	0.396	0.35 (0.15–0.82)	0.017	1.21 (0.30–4.85)	0.768
T3 (≥1.742)	0.28 (0.11–0.68)	0.006	0.01 (0.00–0.85)	0.045	0.15 (0.06–0.42)	0.001	0.23 (0.02–3.48)	0.252
Omega-6 PUFA								
T1 (<10.346)	Ref		Ref		Ref		Ref	
T2 (10.346–17.908)	0.30 (0.16–0.58)	0.001	0.01 (0.00–0.75)	0.042	0.17 (0.09–0.33)	<0.001	0.41 (0.09–1.89)	0.222
T3 (≥17.908)	0.94 (0.35–2.51)	0.894	0.00 (0.00–1.01)	0.050	0.37 (0.12–1.13)	0.081	0.79 (0.04–17.58)	0.868
Linoleic acid								
T1 (<10.263)	Ref		Ref		Ref		Ref	
T2 (10.263–17.621)	0.30 (0.16–0.58)	0.001	0.01 (0.00–0.75)	0.042	0.17 (0.09–0.32)	<0.001	0.41 (0.09–1.89)	0.222
T3 (≥17.621)	1.03 (0.38–2.82)	0.951	0.00 (0.00–1.01)	0.050	0.42 (0.14–1.30)	0.128	0.79 (0.04–17.56)	0.869

Ref, reference; OR, odds ratio; CI, confidence interval; PUFA, polyunsaturated fatty acid; ALA, α-linolenic.

## Discussion

This present study examined the association between PUFA intake and depression symptoms in women experiencing infertility by analyzing data in the NHANES database. It was found that omega-3 PUFA and omega-6 PUFA in T2 were associated with lower odds of depression symptoms, indicating that moderate intake of PUFA was beneficial for depression symptoms. Next, we analyzed the relationship between different types of omega-3 and omega-6 and depression symptoms. The findings showed that ALA intake in T2 and T3 and DOA intake in T2 were related to the lower odds of depression symptoms. In addition, we found that omega-3 PUFA and ALA in T2 or T3 had a relationship with the lower odds of depression symptoms in women with infertility who are experiencing menopause and those who are not or those who are without female hormone use. Omega-6 PUFA and linoleic acid in T2 were associated with decreased odds of depression symptoms in those who are experiencing menopause and those who are not and those who are without female hormone use.

Omega-3 PUFA is considered to be useful in treating several chronic diseases; in particular, some studies emphasized its role in the prevention or treatment of depression (18). The deficiency of omega-3 may cause the impairment of neuronal function (especially serotonin and dopamine neurotransmitters) and a change in inflammatory status (8). In the elderly, the supplementation of omega-3 was found to be a valid low-cost prevention strategy to counteract depression (19). Mocking et al. suggested that omega-3 PUFA should be supplemented to treat postpartum depression (20). The meta-analysis by Liao et al. displayed an overall beneficial effect of omega-3 PUFA on depressive symptoms (11). In this study, we found that moderate intake of omega-3 PUFA was beneficial for depression symptoms in women experiencing infertility. The mechanisms of the antidepressant role of omega-3 PUFA have not been fully understood. Omega-3 PUFA deficiency has been reported to be associated with dysfunctions of serotonin, dopamine, and norepinephrine neurotransmission, which are related to emotional disorders including depression (21). Another potential mechanism of the antidepressant effect of omega-3 PUFA is through regulating neuroinflammation and oxidative stress (22). In individuals with higher levels of oxidative stress, omega-3 PUFA is inversely correlated with depressive symptoms (23). In addition, ALA is a member of the omega-3 PUFA family that cannot be synthesized by humans; therefore, it must be obtained from the diet (24). A study showed that rats that consumed omega-3 PUFA-deficient diets from birth had a higher inflammation level, which was reversed by subsequent feeding with an ALA-containing diet (25). Accordingly, our study found that moderate and higher intake of ALA decreased the odds of depression symptoms in women experiencing infertility.

Omega-6 PUFA has been reported to play a pro-inflammatory role, and a high omega-6 PUFA diet inhibits the anti-inflammatory effect of omega-3 PUFA (26). However, existing evidence shows that elevated intake of omega-6 PUFA does not elevate the level of inflammatory markers and even suggests that omega-6 PUFA may reduce inflammation (26). It was known that inflammation was involved in the onset of depression (8). Thesing et al. reported that there was no relationship between omega-6 PUFA levels and depression (27). Vaz et al. found that higher serum levels of omega-6 PUFA were associated with greater odds of depression in pregnant women (28). In this study, we found that the odds of depression symptoms were lower in women experiencing infertility with the intake of omega-6 PUFA in T2. Furthermore, linoleic acid intake in T2 (10.263–17.621 g) was associated with decreased odds of depression symptoms. The Dietary Guidelines for Americans recommend that the dietary allowance (RDA) for daily linoleic acid is 12 g in adult women (29). Our findings suggested that moderate intake (within the recommended level) of omega-6 PUFA, especially linoleic acid, was beneficial in improving depressive symptoms.

Evidence considered to establish dietary intake recommendations is important to explore the effects of PUFAs on depression in women with infertility. It is recommended by the Dietary Guidelines for Americans that the consumption of n−3 PUFA should be increased by consuming two servings of seafood per week which provides an average of 250 mg/day of n−3 PUFA from marine sources (30). However, in this study, T2 intake of n−3 PUFA (1.150–1.872 g) was linked to a decreased risk of depression symptoms compared with T1 (<1.150 g). The dietary intake of n−3 PUFA in individuals in this study is higher than the recommended intake in more than 60% of the population. Current dietary guidelines for recommended intakes may need to be reassessed, especially for those with specific health problems, such as infertility and depression. There is no scientific rationale for the recommendation of a specific ratio of n−6 PUFA to n−3 PUFA (31). This suggests that more research is needed to determine the optimal intake ratio of n−6 and n−3 PUFA and how this ratio affects the population, including infertile women.

In our study, the majority of the significant associations between PUFA intake and reduced depression risk were observed in the second tertile of intake, rather than the third tertile. The specific cutoff points for tertiles were determined based on the distribution of PUFA intake in the study population. It is possible that the chosen cutoffs may impact the observed associations, leading to the discrepancy between T2 and T3. The sample size within each tertile may have influenced the ability to detect significant associations. If the sample size in Q3 is smaller or the variability is higher, this could affect the statistical power to detect a significant relationship. This pattern suggests that there may be an optimal range of PUFA consumption that is most beneficial for mental health outcomes in women experiencing infertility. These findings highlight the need for more nuanced research into the optimal intake levels of PUFAs for mental health benefits, particularly in the context of infertility.

In this study, we found the association between omega-3 PUFA, ALA, omega-6 PUFA, linoleic acid, and depression symptoms in women with infertility who are experiencing menopause and those who are not and those who are without female hormone use, while the association between PUFA intake and depression symptoms was not found in women experiencing infertility with female hormone use. This may be explained by the fact that the use of female hormones weakened the relationship between PUFA and depression symptoms. The use of female hormones can significantly alter hormone levels, potentially affecting PUFA metabolism. Hormones are found to have impacts on macronutrient metabolism (32). Therefore, hormone therapy could modify how omega-3 and omega-6 PUFAs impact depression symptoms. Hormone therapy could potentially overshadow or negate the positive effects of PUFAs on mood by directly influencing mood (33). Hormonal changes, especially the decrease in estrogen, are believed to contribute to the development of depression (34). Further research is needed to explore the specific mechanisms by which female hormones interact with PUFAs and their impact on mental health.

The findings of our study, which analyzed data from the NHANES database, have several clinical implications. Our results suggest that a moderate intake of PUFAs, particularly omega-3 and omega-6, may be beneficial in managing depression symptoms among women experiencing infertility. This insight can guide healthcare providers in offering dietary advice that emphasizes the inclusion of PUFAs in the daily diets of their patients. The associations found between specific types of PUFAs, ALA and DHA, and a reduced risk of depression symptoms indicate that personalized nutrition plans could be developed. These plans could be tailored to the individual’s PUFA intake levels and their specific health needs, including their menopausal status and hormone use. The observation that PUFA intake is associated with depression symptoms in both pre- and postmenopausal women, as well as in those without hormone use, underscores the importance of considering dietary factors in mental health support for women experiencing infertility. This could lead to more holistic treatment approaches that integrate nutrition with other therapeutic interventions. The results may also inform public health policies aimed at promoting mental health among women experiencing infertility. Encouraging the consumption of PUFAs through public health campaigns and ensuring their availability in food products can have a broader impact on the mental health of this population.

This study includes a nationally representative sample that provides a chance to explore the association between PUFA intake and depression symptoms in women experiencing infertility. Our study’s focus on the association between various types of PUFA intake and the risk of depression in women experiencing infertility addresses a significant gap in the literature. This research provides valuable insights into the dietary factors that may influence mental health in this specific population, which is often overlooked in broader dietary studies. We have taken careful steps to adjust for potential confounding factors in our analysis. By including a range of covariates, we have minimized the risk of bias and provided a more accurate estimation of the relationship between PUFA intake and depression. To further evaluate the applicability of our findings to different segments of the population, we conducted subgroup analyses based on menopausal status and female hormone use. These analyses allowed us to explore whether the relationship between PUFA intake and depression risk varies across these subgroups, providing a more nuanced understanding of how these factors may interact. Our use of a weighted multivariable logistic regression model, along with stepwise regression to select covariates, demonstrates a methodological rigor that strengthens the validity of our conclusions.

However, it is important to acknowledge several limitations inherent in our study. First, the data we analyzed came from individuals from the USA. Dietary habits and cultural practices vary significantly across different countries and regions. The types and amounts of PUFAs consumed can differ based on local food availability, traditional cuisines, and nutritional guidelines, which may influence the relationship between PUFA intake and depression symptoms. There may be genetic and biological differences between populations that could affect the metabolism of PUFAs and their impact on mental health. These variations could lead to different responses to PUFA intake among individuals from different regions. Therefore, the findings of our study may need to be verified in individuals from other countries or regions. Second, this is a cross-sectional study. The primary limitation of cross-sectional studies is their inability to establish a cause-and-effect relationship between variables. Since the data are collected at a single point in time, we can only observe associations, not the direction or sequence of events. This means that while we can report the relationship between PUFA intake and depression symptoms, we cannot definitively state that one causes the other. Cross-sectional studies are susceptible to confounding, where external factors not measured in the study could influence the relationship between the exposure and outcome. Despite our efforts to control for potential confounders, there may still be unmeasured variables that could affect our results. The classification of participants based on their responses at a single point in time may not accurately reflect their long-term status. This misclassification can dilute the observed associations and affect the study’s precision. In the future, longitudinal studies and randomized controlled trials are needed to provide evidence of causality. Third, using data on the 24-h dietary recall and PHQ-9 questionnaire is a limitation. The 24-h dietary recall relies on the participants’ memory and self-reporting of their food intake over the previous day. This method is inherently subjective and can be influenced by various factors, such as social desirability bias, where participants may report healthier eating habits than their actual behavior. The PHQ-9 is a self-administered questionnaire designed to screen for depression symptoms. While it is a widely used and validated tool, its reliance on self-reporting can also be subject to bias. Participants may underreport their symptoms if they are not fully aware of their mental health status or if they wish to present themselves in a more positive light. Conversely, they may overreport symptoms due to heightened self-awareness or a tendency to focus on negative experiences. The 24-h dietary recall captures a single day’s dietary intake, which may not accurately reflect an individual’s long-term eating habits. Daily food consumption can vary significantly, and a single day’s recall may not be representative of the participant’s usual diet. In light of these limitations, we emphasize the need for caution when interpreting our findings. Future research should consider incorporating more objective measures of dietary intake, such as multiple 24-h recalls, food diaries, or biomarkers, as well as more comprehensive assessments of mental health status.

## Conclusion

This study revealed a significant association between moderate consumption of omega-3 and omega-6 PUFAs and a reduced likelihood of experiencing depression symptoms among women experiencing infertility. The findings suggest that dietary recommendations encouraging the inclusion of PUFA-rich foods may be beneficial in managing depressive symptoms in this patient population.

## Data availability statement

The original contributions presented in the study are included in the article/**Supplementary Material**, further inquiries can be directed to the corresponding author.

## Ethics statement

The requirement of ethical approval was waived by Tongji Hospital, School of Medicine, Tongji University for the studies involving humans because the data analyzed in the study were publicly available. The studies were conducted in accordance with the local legislation and institutional requirements. The ethics committee/institutional review board also waived the requirement of written informed consent for participation from the participants or the participants’ legal guardians/next of kin because of the retrospective nature of the study.

## Author contributions

YH: Conceptualization, Funding acquisition, Project administration, Supervision, Writing – original draft, Writing – review & editing. XJ: Data curation, Formal analysis, Investigation, Methodology, Writing – review & editing. LS: Conceptualization, Project administration, Writing – review & editing.

## References

[1] Vander Borght M Wyns C. Fertility and infertility: Definition and epidemiology. Clin Biochem. (2018) 62:2–10. doi: 10.1016/j.clinbiochem.2018.03.012 29555319

[2] Agarwal A Mulgund A Hamada A Chyatte MR. A unique view on male infertility around the globe. Reprod Biol Endocrinol. (2015) 13:37. doi: 10.1186/s12958-015-0032-1 25928197 PMC4424520

[3] Dadhwal V Choudhary V Perumal V Bhattacharya D. Depression, anxiety, quality of life and coping in women with infertility: A cross-sectional study from India. Int J Gynaecol Obstet. (2022) 158:671–8. doi: 10.1002/ijgo.14084 34957556

[4] Wang L Tang Y Wang Y. Predictors and incidence of depression and anxiety in women undergoing infertility treatment: A cross-sectional study. PLoS One. (2023) 18:e0284414. doi: 10.1371/journal.pone.0284414 37053254 PMC10101516

[5] Dong M Xu X Li Y Wang Y Jin Z Tan J. Impact of infertility duration on female sexual health. Reprod Biol Endocrinol. (2021) 19:157. doi: 10.1186/s12958-021-00837-7 34627263 PMC8501599

[6] Fabozzi G Verdone G Allori M Cimadomo D Tatone C Stuppia L. Personalized nutrition in the management of female infertility: new insights on chronic low-grade inflammation. Nutrients. (2022) 14:1918. doi: 10.3390/nu14091918 35565885 PMC9105997

[7] Wang R Feng Y Chen J Chen Y Ma F. Association between polyunsaturated fatty acid intake and infertility among American women aged 20-44 years. Front Public Health. (2022) 10:938343. doi: 10.3389/fpubh.2022.938343 36062133 PMC9428268

[8] Grosso G Galvano F Marventano S Malaguarnera M Bucolo C Drago F. Omega-3 fatty acids and depression: scientific evidence and biological mechanisms. Oxid Med Cell Longev. (2014) 2014:313570. doi: 10.1155/2014/313570 24757497 PMC3976923

[9] Lin PY Chang CH Chong MF Chen H Su KP. Polyunsaturated fatty acids in perinatal depression: A systematic review and meta-analysis. Biol Psychiatry. (2017) 82:560–9. doi: 10.1016/j.biopsych.2017.02.1182 28410627

[10] Osuna E Symington EA Malan L Ricci C Zandberg L Smuts CM. Higher n-3 polyunsaturated fatty acid status during early pregnancy is associated with lower risk for depression at 12 months postpartum: The NuPED study. Prostaglandins Leukot Essent Fatty Acids. (2023) 190:102528. doi: 10.1016/j.plefa.2022.102528 36716632

[11] Liao Y Xie B Zhang H He Q Guo L Subramanieapillai M. Efficacy of omega-3 PUFAs in depression: A meta-analysis. Transl Psychiatry. (2019) 9:190. doi: 10.1038/s41398-019-0515-5 31383846 PMC6683166

[12] Decandia D Landolfo E Sacchetti S Gelfo F Petrosini L Cutuli D. n-3 PUFA improve emotion and cognition during menopause: A systematic review. Nutrients. (2022) 14:1982. doi: 10.3390/nu14091982 35565948 PMC9100978

[13] Kobayashi M Ogawa K Morisaki N Tani Y Horikawa R Fujiwara T. Dietary n-3 Polyunsaturated Fatty Acids in Late Pregnancy and Postpartum Depressive Symptom among Japanese Women. Front Psychiatry. (2017) 8:241. doi: 10.3389/fpsyt.2017.00241 29218019 PMC5703735

[14] NHANES. Available online at: https://wwwn.cdc.gov/nchs/nhanes/Search/default.aspx (Accessed November 3, 2023).

[15] Patel JS Oh Y Rand KL Wu W Cyders MA Kroenke K. Measurement invariance of the patient health questionnaire-9 (PHQ-9) depression screener in U.S. adults across sex, race/ethnicity, and education level: NHANES 2005-2016. Depress Anxiety. (2019) 36:813–23. doi: 10.1002/da.22940 PMC6736700 31356710

[16] Li Y Yuan X Zheng Q Mo F Zhu S Shen T. The association of periodontal disease and oral health with hypertension, NHANES 2009-2018. BMC Public Health. (2023) 23:1122. doi: 10.1186/s12889-023-16012-z 37308938 PMC10262359

[17] Mcclure ST Schlechter H Oh S White K Wu B Pilla SJ. Dietary intake of adults with and without diabetes: results from NHANES 2013-2016. BMJ Open Diabetes Res Care. (2020) 8:e001681. doi: 10.1136/bmjdrc-2020-001681 PMC7590352 33099509

[18] Simopoulos AP. Evolutionary aspects of diet, the omega-6/omega-3 ratio and genetic variation: nutritional implications for chronic diseases. BioMed Pharmacother. (2006) 60:502–7. doi: 10.1016/j.biopha.2006.07.080 17045449

[19] Farioli Vecchioli S Sacchetti S Nicolis Di Robilant V Cutuli D. The role of physical exercise and omega-3 fatty acids in depressive illness in the elderly. Curr Neuropharmacol. (2018) 16:308–26. doi: 10.2174/1570159X15666170912113852 PMC5843982 28901279

[20] Mocking RJT Steijn K Roos C Assies J Bergink V Ruhé HG. Omega-3 fatty acid supplementation for perinatal depression: A meta-analysis. J Clin Psychiatry. (2020) 81:19r13106. doi: 10.4088/JCP.19r13106 32898343

[21] Su KP. Biological mechanism of antidepressant effect of omega-3 fatty acids: how does fish oil act as a 'mind-body interface'? Neurosignals. (2009) 17:144–52. doi: 10.1159/000198167 19190401

[22] Song C Shieh CH Wu YS Kalueff A Gaikwad S Su KP. The role of omega-3 polyunsaturated fatty acids eicosapentaenoic and docosahexaenoic acids in the treatment of major depression and Alzheimer's disease: Acting separately or synergistically? Prog Lipid Res. (2016) 62:41–54. doi: 10.1016/j.plipres.2015.12.003 26763196

[23] Bigornia SJ Harris WS Falcón LM Ordovás JM Lai CQ Tucker KL. The omega-3 index is inversely associated with depressive symptoms among individuals with elevated oxidative stress biomarkers. J Nutr. (2016) 146:758–66. doi: 10.3945/jn.115.222562 PMC4807643 26936135

[24] Ruxton CH Calder PC Reed SC Simpson MJ. The impact of long-chain n-3 polyunsaturated fatty acids on human health. Nutr Res Rev. (2005) 18:113–29. doi: 10.1079/NRR200497 19079899

[25] Mcnamara RK Jandacek R Rider T Tso P Cole-Strauss A Lipton JW. Omega-3 fatty acid deficiency increases constitutive pro-inflammatory cytokine production in rats: relationship with central serotonin turnover. Prostaglandins Leukot Essent Fatty Acids. (2010) 83:185–91. doi: 10.1016/j.plefa.2010.08.004 PMC2993848 20817496

[26] Innes JK Calder PC. Omega-6 fatty acids and inflammation. Prostaglandins Leukot Essent Fatty Acids. (2018) 132:41–8. doi: 10.1016/j.plefa.2018.03.004 29610056

[27] Thesing CS Bot M Milaneschi Y Giltay EJ Penninx B. Omega-3 and omega-6 fatty acid levels in depressive and anxiety disorders. Psychoneuroendocrinology. (2018) 87:53–62. doi: 10.1016/j.psyneuen.2017.10.005 29040890

[28] Vaz JS Kac G Nardi AE Hibbeln JR. Omega-6 fatty acids and greater likelihood of suicide risk and major depression in early pregnancy. J Affect Disord. (2014) 152-154:76–82. doi: 10.1016/j.jad.2013.04.045 23726775 PMC4239694

[29] Dietary Guidelines for Americans. Available online at: https://health.gov/sites/default/files/2019-09/2015-2020_Dietary_Guidelines.pdf (Accessed November 10, 2023).

[30] Mcguire S. U.S. Department of agriculture and U.S. Department of health and human services, dietary guidelines for Americans 2010. 7th edition, Washington, DC: U.S. Government printing office, January 2011. Adv Nutr. (2011) 2:293–4. doi: 10.3945/an.111.000430 PMC3090168 22332062

[31] Aranceta J Pérez-Rodrigo C. Recommended dietary reference intakes, nutritional goals and dietary guidelines for fat and fatty acids: a systematic review. Br J Nutr. (2012) 107:S8–S22. doi: 10.1017/S0007114512001444 22591906

[32] Comitato R Saba A Turrini A Arganini C Virgili F. Sex hormones and macronutrient metabolism. Crit Rev Food Sci Nutr. (2015) 55:227–41. doi: 10.1080/10408398.2011.651177 PMC4151815 24915409

[33] Wium-Andersen MK Jørgensen TSH Halvorsen AH Hartsteen BH Jørgensen MB Osler M. Association of hormone therapy with depression during menopause in a cohort of Danish women. JAMA Netw Open. (2022) 5:e2239491. doi: 10.1001/jamanetworkopen.2022.39491 36318208 PMC9627415

[34] Giannini A Caretto M Genazzani AR Simoncini T. Neuroendocrine changes during menopausal transition. Endocrines. (2021) 2:405–16. doi: 10.3390/endocrines2040036

