# Supplementary material for: Association between polyunsaturated fatty acids and depression in women with infertility: a cross-sectional study based on the National Health and Nutrition Examination Survey
Source: Front Psychiatry. 2024 Jul 2;15:1345815. doi: 10.3389/fpsyt.2024.1345815 (PMC11249730; doi:10.3389/fpsyt.2024.1345815)
Supplement: Supplementary file 1 [file Table_1.docx]

**Supplementary table 1 The selection of co-variables**

| **Variables** | **Univariable analysis** | | **Multivariable analysis** | |
| --- | --- | --- | --- | --- |
|  | **OR (95% CI)** | ***P*** | **OR (95% CI)** | ***P*** |
| Age | 1.04 (1.01-1.06) | 0.010 |  |  |
| Race |  |  |  |  |
| Non-Hispanic White | Ref |  | Ref |  |
| Non-Hispanic Black | 0.62 (0.38-1.04) | 0.070 | 0.38 (0.20-0.73) | 0.005 |
| Mexican American | 0.46 (0.23-0.93) | 0.031 | 0.51 (0.22-1.18) | 0.115 |
| Others | 0.73 (0.37-1.45) | 0.362 | 0.62 (0.32-1.20) | 0.151 |
| Educational level |  |  |  |  |
| Less than high school | Ref |  |  |  |
| High School | 0.69 (0.36-1.32) | 0.256 |  |  |
| Above high school | 0.54 (0.26-1.12) | 0.098 |  |  |
| PIR |  |  |  |  |
| < 1 | Ref |  |  |  |
| ≥ 1 | 0.59 (0.32-1.12) | 0.103 |  |  |
| Unknown | 0.24 (0.08-0.74) | 0.013 |  |  |
| Marital status |  |  |  |  |
| Married & living with partner | Ref |  | Ref |  |
| Never married & divorced & separated & widowed | 3.44 (2.03-5.84) | < 0.001 | 4.66 (2.62-8.28) | < 0.001 |
| Smoking |  |  |  |  |
| No | Ref |  |  |  |
| Yes | 1.99 (1.18-3.34) | 0.010 |  |  |
| Alcohol drinking |  |  |  |  |
| Never | Ref |  | Ref |  |
| Former | 1.97 (0.88-4.40) | 0.097 | 2.46 (1.04-5.82) | 0.041 |
| Continuous | 1.76 (0.83-3.72) | 0.138 | 2.21 (1.03-4.76) | 0.043 |
| Unknown | 4.60 (2.17-9.78) | < 0.001 | 4.95 (2.28-10.79) | < 0.001 |
| Physical activity |  |  |  |  |
| Low level | Ref |  | Ref |  |
| High level | 0.56 (0.35-0.90) | 0.018 | 0.59 (0.34-1.00) | 0.049 |
| Age at menarche | 1.03 (0.93-1.15) | 0.543 |  |  |
| Menopausal status |  |  |  |  |
| No | Ref |  |  |  |
| Yes | 0.99 (0.47-2.10) | 0.982 |  |  |
| Ever pregnant |  |  |  |  |
| No | Ref |  |  |  |
| Yes | 1.88 (0.95-3.74) | 0.071 |  |  |
| Ovariectomy |  |  |  |  |
| No | Ref |  | Ref |  |
| Yes | 6.78 (2.60-17.66) | < 0.001 | 5.83 (2.04-16.67) | 0.002 |
| Hysterectomy |  |  |  |  |
| No | Ref |  |  |  |
| Yes | 4.59 (2.27-9.26) | < 0.001 |  |  |
| Hypertension |  |  |  |  |
| No | Ref |  |  |  |
| Yes | 2.44 (1.52-3.92) | < 0.001 |  |  |
| Diabetes |  |  |  |  |
| No | Ref |  | Ref |  |
| Yes | 2.46 (1.30-4.65) | 0.006 | 3.14 (1.61-6.11) | 0.001 |
| Dyslipidemia |  |  |  |  |
| No | Ref |  |  |  |
| Yes | 1.91 (1.17-3.14) | 0.011 |  |  |
| CVD |  |  |  |  |
| No | Ref |  |  |  |
| Yes | 4.37 (1.93-9.89) | 0.001 |  |  |
| Pelvic infection |  |  |  |  |
| No | Ref |  | Ref |  |
| Yes | 3.74 (1.74-8.04) | 0.001 | 2.52 (1.13-5.59) | 0.024 |
| Female hormones use |  |  |  |  |
| No | Ref |  | Ref |  |
| Yes | 3.26 (1.79-5.93) | < 0.001 | 2.37 (1.29-4.35) | 0.006 |
| Sleep duration |  |  |  |  |
| < 7 | Ref |  |  |  |
| 7-9 | 0.87 (0.52-1.45) | 0.579 |  |  |
| > 9 | 2.27 (1.01-5.09) | 0.047 |  |  |
| BMI |  |  |  |  |
| < 25 | Ref |  |  |  |
| 25-30 | 1.96 (0.98-3.94) | 0.057 |  |  |
| ≥ 30 | 2.78 (1.50-5.15) | 0.002 |  |  |
| Total energy | 1.00 (1.00-1.00) | 0.172 |  |  |
| Total PUFA | 0.99 (0.97-1.01) | 0.449 |  |  |

Ref, Reference; OR, odds ratio; CI, confidence interval; PIR, poverty-to-income ratio; CVD, cardiovascular disease; BMI, body mass index; PUFA, polyunsaturated fatty acids.
